# MLVA and LPS Characteristics of *Brucella canis* Isolated from Humans and Dogs in Zhejiang, China

**DOI:** 10.3389/fvets.2017.00223

**Published:** 2017-12-18

**Authors:** Dongri Piao, Heng Wang, Dongdong Di, Guozhong Tian, Jiantong Luo, Wenjie Gao, Hongyan Zhao, Weimin Xu, Weixing Fan, Hai Jiang

**Affiliations:** ^1^State Key Laboratory for Infectious Disease Prevention and Control, Collaborative Innovation Center for Diagnosis and Treatment of Infectious Diseases, National Institute for Communicable Disease Control and Prevention, Chinese Center for Disease Control and Prevention, Beijing, China; ^2^Hangzhou Center for Disease Control and Prevention (HZCDC), Hangzhou, China; ^3^China Animal Health and Epidemiology Center, Qingdao, China

**Keywords:** MLVA, LPS biosynthesis, *Brucella canis*, humans and dogs, epidemiology

## Abstract

**Background:**

*Brucella canis* is a pathogenic bacterium that causes brucellosis in dogs, and its zoonotic potential has been increasing in recent years. *B. canis* is a rare source of human brucellosis in China, where *Brucella melitensis* has been the major pathogen associated with human brucellosis outbreaks. In late 2011, a case of a *B. canis* infection was detected in a human patient in Zhejiang Province, China. To compare the genotypes between strains of *B. canis* isolated from the patient and from dogs, a multiple-locus variable-number tandem-repeat analysis (MLVA-16) was performed. In addition, the lipopolysaccharide-synthesis-related genes were analyzed with the *B. canis* reference strain RM6/66.

**Results:**

32 *B. canis* strains were divided into 26 genotypes using MLVA-16 [Hunter-Gaston Diversity Index (HGDI) = 0.976]. The HGDI indexes for various loci ranged between 0.000 and 0.865. All four Hangzhou isolates were indistinguishable using panel 1 (genotype 3) and panel 2A (genotype 28). However, these strains were distinctly different from other isolates from Beijing, Jiangsu, Liaoning, and Inner Mongolia at Bruce 09. The emergence of a human *B. canis* infection was limited to an area. Comparative analysis indicated *B. canis* from canines and humans have no differences in lipopolysaccharide-synthesis locus.

**Conclusion:**

The comprehensive approaches have been used to analyze human and canine *B. canis* isolates, including molecular epidemiological and LPS genetic characteristics. Further detailed analysis of the whole genomic sequencing will contribute to understanding of the pathogenicity of *B. canis* in humans.

## Introduction

Brucellosis is one of the most common zoonotic diseases worldwide. In north of China, brucellosis is a serious endemic disease mainly caused by *Brucella melitensis* infection (biovars 1 and 3). Currently, there were 95 human brucellosis surveillance counties nationwide in order to grasp the epidemiological features. In recent years, a few papers about canine brucellosis have published (1–5). The new available data of canine brucellosis showed the outbreak trends in Beijing and other provinces (6–9).

Human brucellosis infection from *Brucella canis* is not common, probably because *B. canis* is rough lipopolysaccharide devoid of the O-side. Indeed, in *Brucella* spp., the smooth lipopolysaccharide, is highly correlated with pathogenesis (10). Some classical *Brucella* strains, as well as the recently isolated new species, express a smooth phenotype (11, 12). It has been shown that the smooth LPS might play an important role in the invasion and intracellular multiplication of *Brucella* spp. (13). Several LPS biosynthesis genes have been recognized, most of them clustered in the *wbk* and *wbo* genetic regions (14). However, *B. ovis* is naturally devoid of the *wbo* region (15). On the other hand, the *wbk* cluster is present in both the *B. ovis* and the *B. canis* (16).

*Brucella melitensis* was the predominant species associated with humans and animals brucellosis outbreaks in China (9). However, *B. canis* infection cannot be ignored. The epidemiological data of human canine brucellosis and etiology study have been limited in China. *B. canis* is known to infect humans, yet only a few cases have ever been reported. On November 10, 2011, a 45-year-old woman who developed fever, back pain, and fatigue was diagnosed with pleurisy at the Jiaxing People’s Hospital, Zhejiang, China. The rough rose bengal plate agglutination test (RRBT) and rough standard tube agglutination test (RSAT) assays were positive. *B. canis* was also isolated from the blood culture. Because of scarce *B. canis* infection cases in humans, any potential genetic diversity of the *B. canis* strain isolated from different hosts should be investigated. Therefore, the aim of this study was to evaluate the epidemiological relatedness among Chinese *B. canis* strains isolated from dogs and humans. First, variable number of tandem repeats assay was performed to assess the diversity from the different regions, especially in the same geographical area, Zhejiang province. Second, 21 LPS-synthesis related genes were amplified and sequenced, in order to detect genetic mutations between human and canine *B. canis* strains. This comparative analysis will contribute to knowledge of the *Brucella* virulence and microevolution.

## Materials and Methods

### Ethics Approval and Consent to Participate

This study is a retrospective investigation of historical collections strains using modern typing methods and study protocol was approved by the Ethics Committees of National Institute for Communicable Disease Control and Prevention, Chinese Center for Disease Control and Prevention. Informed consent was obtained from the patient before diagnosis in the study. We isolated *Brucella* spp. from patient’ blood sample with his agreement.

### Human *B. canis* Infection Detection

For detection of antibodies against rough *Brucella* spp., the RRBT and RSAT were performed as previously described (17). Blood culture was performed by biphasic blood bottle (BioMérieux Industry, France) by following the manufacturer’s protocol. 7 days later, some colonies were found in solid phase and inoculated into *Brucella* Agar Medium.

### *B. canis* Identification and DNA Preparation

Species identification was determined on the basis of phenotype identification procedure (18). 32 *B. canis* strains isolated from Zhejiang, Beijing, Jiangsu, Liaoning, and Inner Mongolia between 1986 and 2011 were included. The genomic DNA was extracted with the DNeasy Blood & Tissue Kit (Qiagen China Ltd., China) by following the manufacturer’s protocol. Species-level identification was performed using Suis-ladder PCR and real-time PCR assay (19, 20). The procedure of *Brucella* isolates were carried out in biosafety level 3.

### MLVA Typing and Data Analysis

The multiple-locus variable-number tandem-repeat analysis (MLVA-16) was performed as previously described (21–23). Sizes of PCR products were evaluated by capillary electrophoresis on an ABI Prism 3130 automated fluorescent capillary DNA sequencer (Applied Biosystems). Genomic DNA from reference strain *B. canis* RM6/66 (NC_010103.1 and NC_010104.1) was used as positive control. The cluster analysis was performed using the UPGMA algorithm (Unweighted Pair Group Method Algorithm) with Euclidean distance matrices. Hunter and Gaston diversity indexes were calculated^1^. Web-based MLVA database^2^ was utilized to compare different strains.

### LPS-Synthesis Characterization of *Brucella* Strains: PCR Assay and Sequence Analysis

21 LPS-synthesis related genes were analyzed. Primers and amplification were previously described (13). The resultant PCR products were purified and sequenced at Qingke Biosciences Company (Beijing, China). The sequence data were compared by web-based BLAST.^3^ Reference strain *B. melitensis* 16M sequences was available in GenBank (AE008917 and AE008918).

### LPS Extraction and SDS-Polyacrylamide Gel Electrophoresis (PAGE)

The LPS was extracted using Lipopolysaccharide Extraction Kit (iNtRON Biotechnology Ltd., Korea) by following the manufacturer’s protocol. SDS-PAGE was performed as previously described (24).

## Results

### Identification of Human Brucellosis by Serological Tests

**Figure 1:**
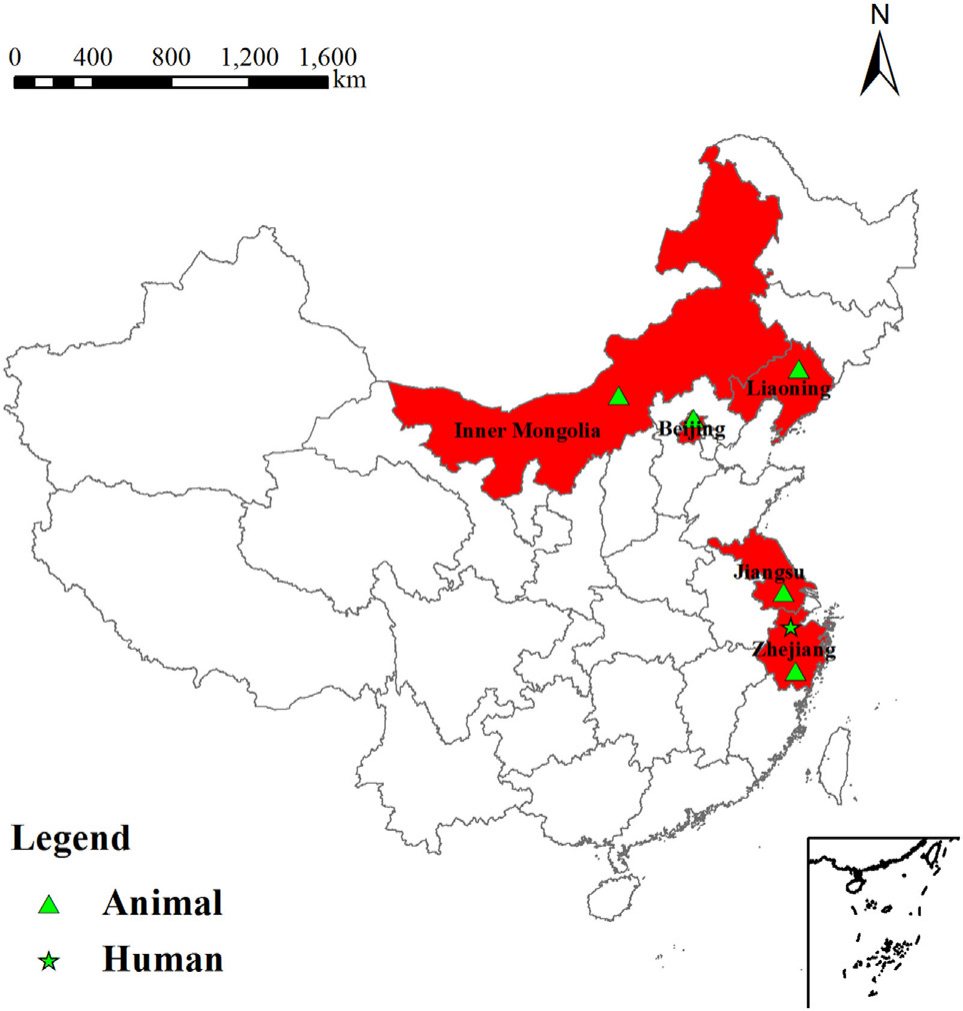
Location of *Brucella canis* isolated from human and animals between 2006 and 2011.

The serum of the patient was tested by RRBT and RSAT tests for anti-R antibodies, and yielded positive results for antibodies against rough *Brucella* spp. Blood culture was performed by biphasic blood bottle. A *B. canis* strain was isolated and showed the same phenotypic characteristics with *B. canis* RM6/66. The location of *B. canis* isolated from human and animals between 2006 and 2011 was shown in Figure 1.

### MLVA Analysis of Human and Canine *B. canis* Strains

**Figure 2:**
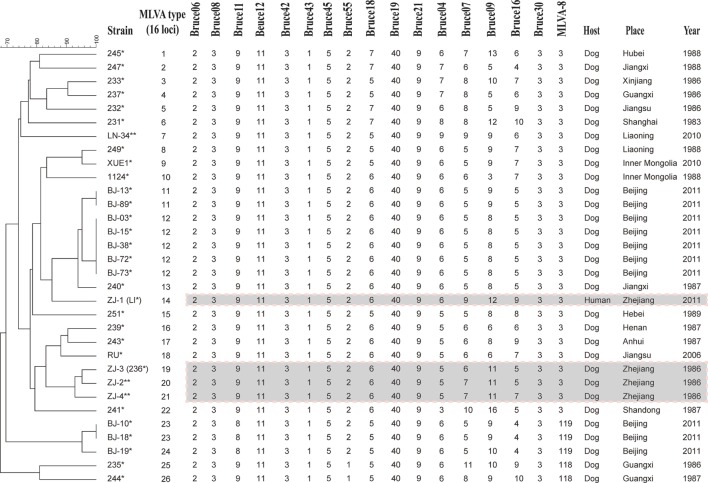
Dendrogram based on the MLVA-16 genotyping assay showing relationships of the four *Brucella canis* isolates from Hangzhou in this study to other *B. canis* strains. Strain: strain name in the laboratory in which the DNA extraction was done; year/place: year and country of isolation (when known); * indicates these strains were previously analyzed in Di et al. (23). ** indicates these strains have been analyzed in this study.

32 *B. canis* strains were grouped into 26 genotypes using MLVA-16 [Hunter-Gaston Diversity Index (HGDI) = 0.976]. The HGDI indexes ranged between 0.000 and 0.865 (Table 1; Figure 2). 11 loci were of low discriminatory power (HGDI < 0.2). Bruce 09 and Bruce 16 displayed high discriminatory power (HGDI > 0.8), which contained 10 and 7 alleles, respectively. Comparative genetic profiles by MLVA-16 indicated that MLVA-8 and MLVA-11 genotypes of human *B. canis* strain were identical to *B. canis* isolates from dogs. Exception of bruce30 loci, four loci (bruce04, bruce07, bruce09, and bruce16) of panel 2B showed diversity (Table 2).

**Table 1:** Hunter-Gaston Diversity Index (HGDI) for the 32 Chinese *Brucella canis* isolates.

Locus	No. of alleles	HGDI^a^	CI 95%^b^
MLVA-16	26	0.976	0.945–1.000
MLVA-8	3	0.284	0.092–0.476
Bruce06	1	0.000	0.000–0.194
Bruce08	1	0.000	0.000–0.194
Bruce11	2	0.175	0.011–0.340
Bruce12	1	0.000	0.000–0.194
Bruce42	1	0.000	0.000–0.194
Bruce43	1	0.000	0.000–0.194
Bruce45	1	0.000	0.000–0.194
Bruce55	2	0.121	0.000–0.268
Panel 2A	3	0.603	0.506–0.699
Bruce18	3	0.603	0.506–0.699
Bruce19	1	0.000	0.000–0.194
Bruce21	1	0.000	0.000–0.194
Panel 2B	26	0.976	0.945–1.000
Bruce04	6	0.607	0.451–0.763
Bruce07	7	0.758	0.649–0.867
Bruce09	10	0.865	0.803–0.927
Bruce16	7	0.821	0.747–0.894
Bruce30	1	0.000	0.000–0.194

*^a^Hunter and Gaston index*.

*^b^Precision of the diversity index, expressed as 95% upper and lower boundaries*.

**Table 2:** MLVA-8 and MLVA-11 genotypes and numbers of tandem-repeat units for panel 2B loci in *Brucella canis* isolates from Zhejiang province and other regions by MLVA-16.

Strain	Host	Year	MLVA-8 genotype	MLVA-11 genotype	Panel 2B				
					Bruce04	Bruce07	Bruce09	Bruce16	Bruce30
ZJ-1 (LI*)	Human	2011	3^a^	28	6	9	12	9	3
ZJ-2**	Dog	1986	3^a^	28	5	7	11	5	3
ZJ-3 (236*)	Dog	1986	3^a^	28	5	6	11	5	3
ZJ-4**	Dog	1986	3^a^	28	5	7	11	7	3
RU*	Dog	2006	3^a^	28	5	6	6	7	3
LN-34**	Dog	2010	3^a^	26	9	9	9	6	3
XUE1*	Dog	2010	3^a^	26	6	5	9	7	3
BJ-03*, BJ-15*, BJ-38*, BJ-72*, BJ-73*	Dog	2011	3^a^	28	6	5	8	5	3
BJ-13*, BJ-89*	Dog	2011	3^a^	28	6	5	9	5	3
BJ-10*, BJ-18*	Dog	2011	New^b^	26	6	5	9	4	3
BJ-19*	Dog	2011	New^b^	26	6	5	10	4	3

*^a^Genotype 3 (2-3-9-11-3-1-5-2)*.

*^b^New genotype (2-3-8-11-3-1-5-2)*.

* indicates these strains have been analyzed in Di et al. (23).

** indicates these strains have been analyzed in this study.

### Genetic Characteristics of Human and Canine *B. canis* LPS

**Figure 3:**
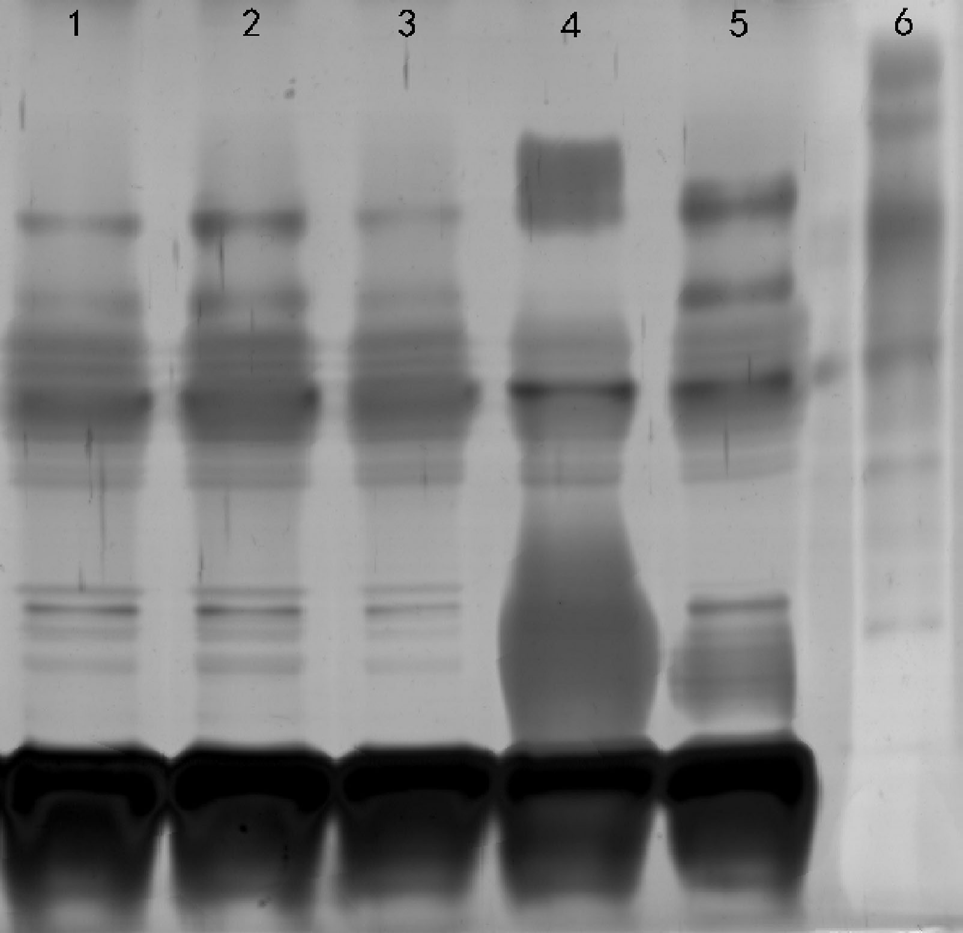
SDS-PAGE analysis of LPS. Lane 1, *Brucella canis* ZJ-1 (human); lane 2, *B. canis* ZJ-2 (dog); lane 3, *B. canis* RM6/66; lane 4, *Brucella melitensis* 16M; lane 5, *B. suis* 1330S; lane 6, molecular weight marker.

21 LPS-synthesis genes were analyzed with the reference strain *B. canis* RM6/66. Comparative analysis indicated *B. canis* from canines and humans had no differences in LPS-synthesis locus. We next sought to extend the LPS-synthesis genetic characterization between *B. canis* isolated from human and canines. We also extracted the LPS and performed SDS-PAGE analysis. The results revealed no difference was found between *B. canis* isolated from human and canines (Figure 3).

## Discussion

*Brucella canis* infection was first reported in China in 1984 (6). A nationwide survey on the *B. canis* infection was conducted in 25 provinces. Epidemic regions were primarily located in south and southeast China during the 1970s and 1980s (6). In 2011, a *B. canis* outbreak was investigated in a beagle dog breeding farm in Beijing. 48.75% (39/80) was positive by RSAT (7). On November 10, 2011, a 45-year-old woman who developed fever, back pain, and fatigue was diagnosed with pleurisy at the Jiaxing People’s Hospital, Zhejiang, China. Although the epidemiological contact history, brucellosis symptoms were atypical. The RRBT and RSAT tests for anti-R antibodies yielded positive results, considered to be evidence of *B. canis* infection. On December 12, the patient blood was sent to the Zhejiang Center for Disease Prevention and Control. After several days, an isolate was collected from the blood culture. This isolate displayed rough colony using acridine orange staining. It also showed strong agglutination with anti-R monospecific sera and weak agglutination with anti-*Brucella* monospecific sera. Surveillance data showed most human brucellosis cases were caused by *B. melitensis* species (8, 9). There were few reported human brucellosis cases caused by *B. canis* species. A detailed understanding of genetic diversity of circulating *B. canis* strains in animals and humans should be achieved to provide appropriate prevention measures for subsequent eradication programs.

Hangzhou city is low prevalence of brucellosis in domestic animals and human populations. We discovered that four *B. canis* strain were classified into genotype 3 by MLVA-8 analysis. As subset panel 2A loci showed no diversity, the genotypic polymorphism was solely due to the panel 2B loci (Table 2). The lack of genetic diversity in panel 1 and panel 2A suggests that these *B. canis* strains are highly homogenous. Further analysis showed the three dog MLVA genotypes were only a single-locus variant or double-locus variants in bruce07 (panel 2B) and bruce21 (panel 2A), respectively. However, the human isolate shared only bruce30 (panel 2B) from the dog isolates, which originated from the same breeding farm. These minor mutants probably reflect adaptation or microevolution (21). Although the three dog isolates analyzed in this study were from 25 years earlier, with no reported intermediate infection, it is of interest that the time-distant isolates showed greater homology than geographically distant isolates, consistent with local persistent transmission. It may suggest a recent evolution from a common ancestor in Hangzhou.

Additionally, these four *B. canis* isolates were compared with some dog isolates from other China provinces: Beijing, Jiangsu, Liaoning, and Inner Mongolia, between 2006 and 2011. Diversities were found with MLVA-16. Previously we observed that *B. canis* isolates from dogs either separate into a known genotype 3 or a new genotype with MLVA-8 (7). Interestingly, the Hangzhou strains were distinctly different from the above-mentioned strains because of the bruce09. It could be deduced that the Hangzhou strains may have originated from different restricted geographic region compared with the other strains. The differences may be explained by the bacterial adaptation to host and environment that produce genetic changes or polymorphisms. Zhejiang province was an epidemic region where *B. melitensis* was the dominant epidemic species and *B. abortus* was an accessory species (unpublished). However, *B. canis* infection has complicated the current epidemic situation. Nationwide, the true incidence of *B. canis* in humans remains unknown; therefore, sero-prevalence survey data are needed to determine the risk of *B. canis* infections. The human canine brucellosis was limited to one area, which is useful in monitoring the source of infection when outbreaks occur in other areas free canine brucellosis.

For LPS-synthesis-related genes, no mutations were detected among these strains, demonstrating the presence of the same LPS genetic loci responsible for rough morphology. We also extracted LPS and performed SDS-PAGE analysis. But, no difference was found between *B. canis* isolated from human and canine. Importantly, the human *B. canis* strain is a precious resource for studying *Brucella* pathogenicity. The whole genome sequencing of the human *B. canis* strain is ongoing and could be useful for the development of diagnostic tools, in order to reduce health complications of this infection in dogs and humans.

## Conclusion

For the first time, comprehensive approaches have been used to analyze human and animal *B. canis* isolates with molecular epidemiological and genetic characteristics. Further detailed analysis of the whole genomic sequencing will contribute to understanding of the pathogenicity of *B. canis* in human.

## Ethics Statement

After approval by the ethics committee, we included patient data in this study (Ethics Committee, National Institute for Communicable Disease Control and Prevention, Chinese Center for Disease Control and Prevention).

## Author Contributions

HJ and HW designed this study and did most of the typing work. HW, JL, WX, and WG were in charge of the epidemiological investigation and collection of Hangzhou strains. GT, HZ, and DP prepared the DNA samples. HW and DP wrote the report. All authors read and approved the final manuscript. HJ and WF are guarantors of the paper.

## Conflict of Interest Statement

The authors declare that the research was conducted in the absence of any commercial or financial relationships that could be construed as a potential conflict of interest.

## Footnotes

^1^http://www.hpa-bioinformatics.org.uk/cgi-bin/DICI/DICI.pl.

^2^http://mlva.u-psud.fr/.

^3^http://blast.ncbi.nlm.nih.gov/Blast.cgi/.
